# Diagnosis and treatment of spontaneous intracranial hypotension due to cerebrospinal fluid leakage

**DOI:** 10.1186/s40064-016-3775-z

**Published:** 2016-12-22

**Authors:** Yake Zheng, Yajun Lian, Chuanjie Wu, Chen Chen, Haifeng Zhang, Peng Zhao

**Affiliations:** 1 Department of Neurology, The First Affiliated Hospital of Zhengzhou University, 1 Jianshe East R, Zhengzhou, 450052 Henan People’s Republic of China; 2 The First People’s Hospital of Zhengzhou, Zhengzhou, China

**Keywords:** Intracranial hypotension, Postural headache, CSF, Myelography

## Abstract

**Introduction:**

Spontaneous intracranial hypotension is one of the causes of a postural headache in young people. In this study, the diagnosis and treatment results of a case of intracranial hypotension headache due to spinal cerebrospinal fluid leakage were reported. Up to now, there is not absolutely effective treatment for intracranial hypotension headache.

**Case description:**

A 32-year-old woman complained, a headache after prolonged sitting that presented with nausea; vomiting; increased pain during walking; and decreased or absent pain after lying down. The dramatic improvement of this cephalalgia with epidural blood patch treatment confirmed the diagnosis.

**Discussion and Evaluation:**

To the best of our knowledge, this is the first reported of radiographic contrast before and after epidural blood patch. Improved clinical diagnosis and treatment of spontaneous intracranial hypotension. The patient didn't feel any discomfort, no complications such as infection etc. were observed. A small dose of intrathecal gadolthis is the first reported case ofinium during CEMRM allows for improved detection of CSF leakage.

**Conclusions:**

Leakage of spinal CSF is a major cause of spontaneous intracranial hypotension. In order to improve clinical diagnosis and provide effective treatment, the precise etiology of spontaneous intracranial hypotension should be investigated in each patient.

## Background

Spontaneous intracranial hypotension, which is commonly caused by the leakage of spinal cerebrospinal fluid (CSF), is one cause of postural headache in young people. The incidence of spontaneous intracranial hypotension is low, with international literature reporting a rate of only 5/10,000 (Gordon 2009). Patients may experience cranial nerve damage, ataxia, increased muscle tone, personality changes, and even disturbance of consciousness.

In the present report, we discuss the diagnosis and treatment of a patient with intracranial hypotension headache due to spinal CSF leakage, providing a basis for the clinical diagnosis and treatment of spontaneous intracranial hypotension headache.

## Case presentation

A 32-year-old woman was admitted to our hospital with the primary complaint of a persistent headache. Twenty-five days prior to admission, she experienced a headache after prolonged sitting; the headache involved a persistent, explosion-like pain in the bilateral temporal lobes and the top of head, which increased during walking and decreased or was absent when lying down, and was accompanied by nausea and vomiting. A lumbar puncture was performed 10 days after admission in order to measure cerebrospinal fluid (CSF) pressure. Lumbar puncture results revealed that the CSF was light yellow in color and slightly cloudy, with a pressure of 60 mmH_2_O, leukocyte count of 10 × 10^6^, and protein level of 600 mg/L (Table 1). The patient was diagnosed with intracranial hypotension headache and was given fluid replacement and bed rest. Two days later, intracranial pressure was again measured via lumbar puncture and was found to be 95 mmH_2_O. At this point, the CSF was yellow in color and slightly cloudy, with a leukocyte count of 8 × 10^6^, and protein level of 1000 mg/L. The headache symptoms resolved after 1 week of continuous treatment. However, 2 days later, the postural headache reappeared. The CSF pressure at this time was 30 mmH_2_O, with a cell count of 13 × 10^6^ and protein level of 1862.2 mg/L (Table 1).

**Table 1:** Cerebrospinal fluid results of patients with lumbar puncture

Date of lumber puncture	Intracranial pressure (mmH_2_O)	Conventional test	Biochemical test (mg/L)
2015.11.6	60	Light yellow, slightly cloudy	600
2015.11.8	95	Light yellow, slightly cloudy	1000
2015.11.18	30	Light yellow, slightly cloudy	1862.2
2015.11.21	35	Colorless and transparent, leukocyte count: 4 × 10^6^	1146
2015.12.3	90	Colorless and transparent, leukocyte count: 5 × 10^6^	407

Brain magnetic resonance imaging (MRI) and computed tomography (CT) results indicated the presence of a subdural hematoma. The patient had no history of hypertension, diabetes, trauma, or surgery. Examination upon admission revealed the following results: her facial expression indicated pain, she was conscious, had normal-to-advanced intelligence, normal cranial nerve function, normal limb muscle strength, no bilateral pathological characteristics, and no meningeal irritation. The results of post-admission physical examinations [routine blood tests, electrolytes, kidney function, liver function, blood coagulation tests (four items), vascular inflammatory markers, immune function tests, etc.] were all normal. On the second day after admission, lumbar puncture revealed that the CSF was colorless and transparent, with an intracranial pressure of 35 mmH_2_O, a leukocyte count of 4 × 10^6^, and a protein level of 1146 mg/L (Table 1). Spinal MRI revealed no abnormalities.

**Fig. 1:**
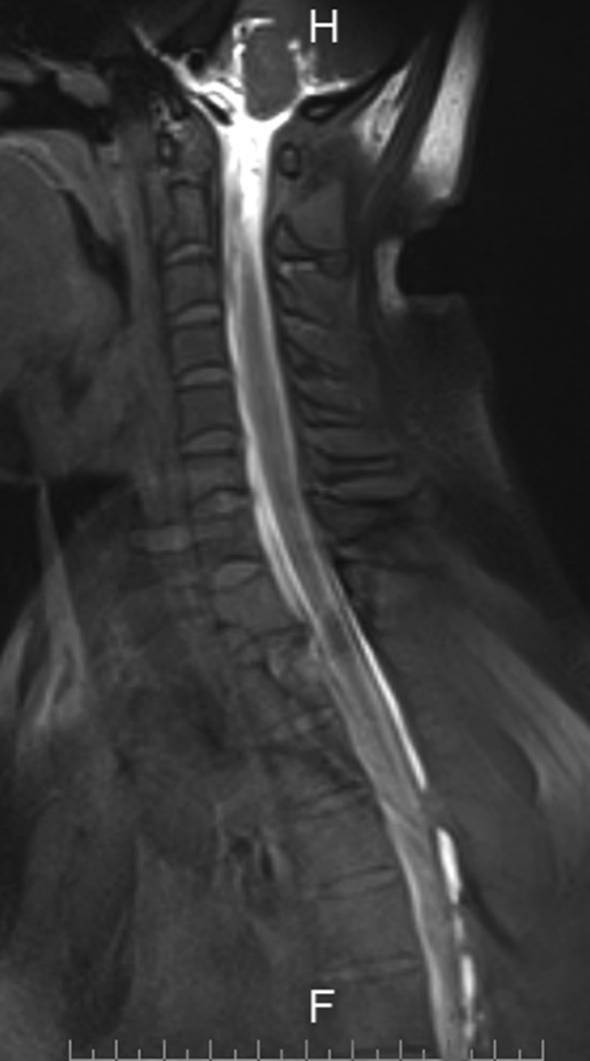
Contrast agent leakage at the level of 3rd–6th thoracic vertebra in sagital

**Fig. 2:**
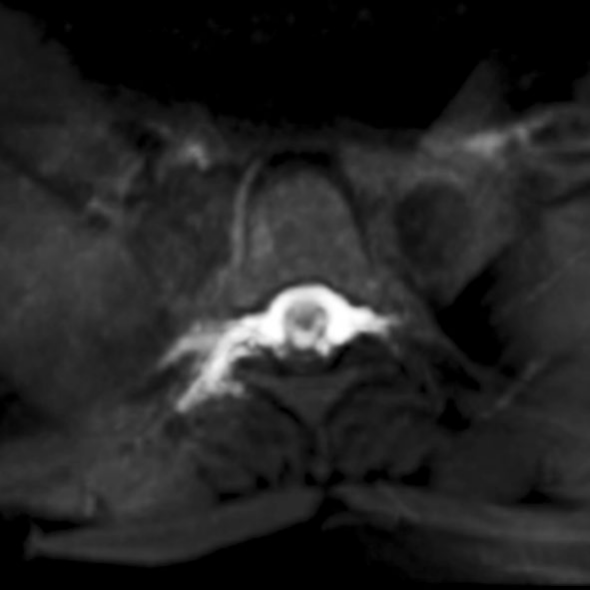
Contrast agent leakage at the level of 3rd–6th thoracic vertebra in coronal position

On the sixth day after hospital admission, the patient underwent contrast-enhanced magnetic resonance myelography (CEMRM). The specific procedure was conducted as follows: after successful lumbar puncture, 10 mL of 5% gadoterate meglumine contrast agent was slowly injected into the spinal subarachnoid cavity at the intervertebral space between the third and fourth lumbar vertebrae, following which 10 mL of saline was slowly injected into the same region. MR images (2D TSE Dixon T1) of the brain, thoracic neck region, and lumbar spine were obtained 15 min after contrast injection. The images indicated leakage of the contrast agent at the level of the third to sixth thoracic vertebrae (Figs. 1, 2). The patient was diagnosed with spontaneous intracranial hypotension headache, according to the updated diagnostic criteria revised by Schievink et al. (2011).

**Fig. 3:**
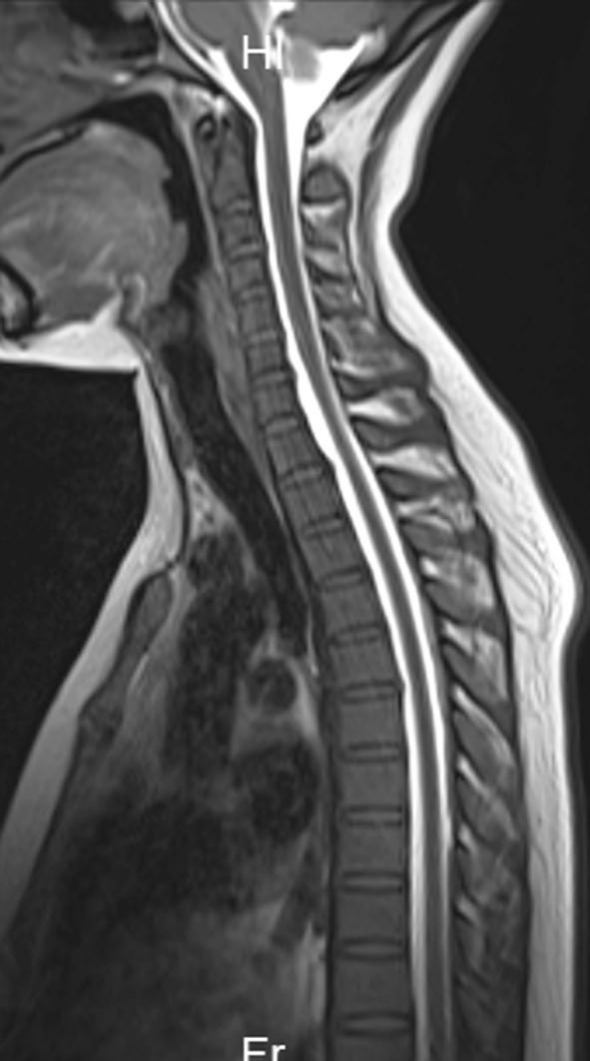
Images of spinal angiography with gadoterate meglumine at the level of 3rd–6th thoracic vertebra conducted 7 days after treatment, with no contrast agent leakage being found in sagital

**Fig. 4:**
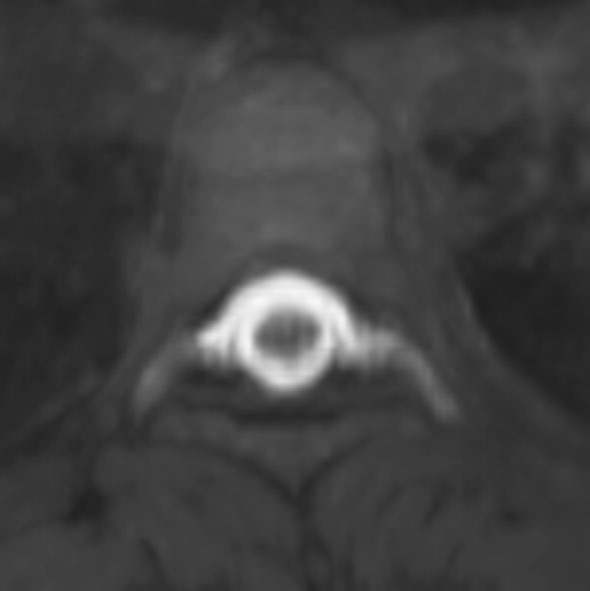
Images of spinal angiography with gadoterate meglumine at the level of 3rd–6th thoracic vertebra conducted 7 days after treatment, with no contrast agent leakage being found in coronal position

On the patient’s seventh day of hospital admission, an epidural blood patch was performed at the level of the fourth thoracic vertebra. An epidural injection was performed, and 15 mL of autologous blood was injected. The patient reported experiencing no discomfort after the procedure. After treatment, the patient was placed on 48 h of bed rest and experienced no headache when getting out of bed thereafter. Seven days after the epidural blood patch, the patient again underwent spinal angiography, which revealed no leakage of contrast agent (Figs. 3, 4). The intracranial pressure was 90 mmH_2_O. The condition of the CSF was normal; it was colorless and transparent, with a leukocyte count of 6 × 10^6^, and a protein level of 407 mg/L. The patient experienced complete remission of symptoms and was then discharged. Follow-up was conducted 1 month after discharge, by which time the patient had experienced no recurrence of symptoms.

## Discussion

The most common cause of spontaneous intracranial hypotension headache is spinal CSF leakage, but the underlying mechanisms remain unknown. It is thought to be related to connective tissue diseases. Indeed, more than 20% of patients with CSF leakage have Marfan syndrome, and abnormal expression of fibrous proteins has been noted in some patients. Research has also indicated that minor trauma to the weak parts of the dura may lead to CSF leakage (Schievink 2006). However, the patient presented here had neither a history of connective tissue disease nor trauma, and reported no relevant family history. Although brain MRI revealed the presence of a subdural hematoma, no significant downward displacement of the brain tissues was observed, it may have been related to the short disease duration and the recumbent position adopted by the patient with the onset of symptoms.

A small dose of intrathecal gadolinium during CEMRM allows for improved detection of CSF leakage (Albayram et al. 2008). A literature review of reports published between May 2007 and April 2013 indicated that the rate of detection of CSF leakage by means of CT myelography is 13%, while that using CEMRM is 38% (Chazen et al. 2014). The patient in the present case experienced recurrent intracranial hypotension headache, although CSF leakage was not detected when using conventional spinal cord MRI. However, after subarachnoid injection of the gadolinium-based contrast agent, the contrast-enhanced MRI scan revealed spinal extravasation at the level of the third to sixth thoracic vertebrae. The patient underwent several lumbar puncture procedures (L2, L3), although no leakage of contrast agent was observed at the lumbar level after intrathecal injection of the contrast agent. Therefore, CSF leakage due to repeated lumbar puncture was not considered to be a cause for intracranial hypotension headache in this case.

Treatment of spontaneous intracranial hypotension headache aims to prevent the leakage of CSF, and to restore CSF volume and brain buoyancy (Schievink 2000). Treatments are commonly conservative in nature and include bed rest (Berroir et al. 2004), abundant fluid replacement, and the use of an abdominal binder (Mokri 2010). Other treatments, including steroids, intravenous injection of caffeine and theophylline, and oral administration of acetazolamide, remain controversial (Mokri 2010). Therefore, the patient reported here was treated conservatively, although fluid replacement and bed rest were not sufficient to relieve her symptoms. Therefore, improved therapeutic interventions are required in order to resolve such symptoms and prevent further complications.

An epidural blood patch is a therapeutic procedure in which an appropriate amount of autologous blood is injected into the epidural space, resulting in instant relief of symptoms in 90% of patients (Ferrante et al. 2010). The success rate for injections of 10–15 mL is 80%, while the success rate of 20-mL injections is greater than 95% (Allegri et al. 2010). Although there is no standard dose for injection, alleviation of symptoms occurs almost immediately, and repeated injections may be performed if the symptoms are not alleviated by the first injection.

Reported in both domestic and international literature, blood patch therapy can be performed at either the lumbar level or at the site of extravasation. We used the latter method, considering that the success rate of autologous blood injection in the peripheral area of the extravasation site is higher than that for the former method. Furthermore, reports have indicated that treatment success could be achieved by performing the blood patch at the extravasation site in patients for whom procedures at the lumbar level have been unsuccessful (Wang et al. 2009). The patient presented here was re-examined using gadolinium-based CEMRM 7 days after treatment, at which time no further leakage of the contrast agent occurred, suggesting that the blood patch therapy was effective. Seven days after blood patch therapy, spinal myelography revealed that leakage of the contrast agent at the third–sixth thoracic vertebrae (extravasation site) had resolved. Previous reports have not discussed the performance of spinal myelography after treatments, although we believe that the success rate of blood patch therapy at the site of extravasation is relatively high. As long as the standard procedures are followed, the risk is relatively small.

The causes of spontaneous intracranial hypotension remain unclear, although many cases seem to be associated with trauma, surgery, infection, etc. In the present report, we located the site of extravasation using gadolinium-based MRI myelography. Our findings suggest that epidural blood patch therapy at this site is safe, effective, and exhibits high operability and repeatability. After treatment, gadolinium-based MRI myelography was repeated, which showed that leakage of the contrast agent had resolved at the original site of extravasation. However, further studies utilizing larger samples are required to increase the detection rate of spontaneous intracranial CSF leakage, and to enhance the safety and efficacy of treatment.

## Conclusion

Leakage of spinal CSF is a major cause of spontaneous intracranial hypotension. In order to improve clinical diagnosis and provide effective treatment, the precise etiology of spontaneous intracranial hypotension should be investigated in each patient.

## Abbreviations

CEMRM: contrast-enhanced magnetic resonance myelography
CSF: cerebrospinal fluid
CT: computed tomography
MRI: magnetic resonance imaging
